# Transcranial Magnetic Stimulation Improves Brain Network Functional Connectivity in a patient with Posterior Cortical Atrophy

**DOI:** 10.1002/alz70858_106629

**Published:** 2025-12-26

**Authors:** Anna Du, Thiago Paranhos, Bonnie Wong, Nneka Watson, Daisy Hochberg, Megan Quimby, Yuta Katsumi, Brad C. Dickerson, Mark C. Eldaief, Alexandra Touroutoglou

**Affiliations:** ^1^ Frontotemporal Disorders Unit and Massachusetts Alzheimer's Disease Research Center, Department of Neurology, Massachusetts General Hospital and Harvard Medical School, Boston, MA, USA; ^2^ Frontotemporal Disorders Unit, Department of Neurology, Massachusetts General Hospital and Harvard Medical School, Boston, MA, USA

## Abstract

**Background:**

Posterior cortical atrophy (PCA) is a clinical syndrome characterized by visuospatial and visuoperceptual symptoms arising from neurodegeneration of the posterior temporoparietal and occipital cortices. Focal neuromodulation such as repetitive transcranial magnetic stimulation (rTMS) can change resting‐state functional connectivity in a network‐specific manner.

**Objective:**

To investigate whether intermittent theta burst stimulation (iTBS) (a form of rTMS), can selectively modulate resting‐state functional connectivity (FC) in posterior nodes of the default mode network (DMN) in a patient with PCA with amnestic symptoms.

**Methods:**

A stimulation target within the left caudal middle frontal (cMFG) was defined based on the maximum FC with the DMN. This target was then stimulated with iTBS in a double‐blind placebo‐controlled paradigm. Changes in the FC of the stimulation target was used as the outcome measure.

**Results:**

Following active stimulation, the patient exhibited selective increase in functional connectivity between the stimulation target and the DMN.

**Conclusion:**

This case report provides novel evidence that network rTMS targeting using individualized resting‐state fMRI can increase network FC in a patient with PCA. Larger controlled trials are needed to determine the impact of neuromodulation on cognitive performance in PCA and to further explore changes in DMN functional connectivity as a mechanism of these effects.

